# Utilization of traditional herbal medicine formulas for unexplained female infertility in Korea: a retrospective study

**DOI:** 10.1186/s12906-023-04192-5

**Published:** 2023-10-19

**Authors:** Su-Ji Choi, Dong-Il Kim

**Affiliations:** 1 Department of Obstetrics and Gynecology, College of Korean Medicine, Dong-eui University, 52-57 Yangjeong-ro, Busanjin-gu, Busan, 47227 Republic of Korea; 2 Department of Obstetrics and Gynecology, College of Korean Medicine, Dongguk University, 27 Dongguk-ro, Ilsandong-gu, Goyang-si, Gyeonggi-do 10326 Republic of Korea

**Keywords:** Infertility, Subfertility, Herbal medicine, Unexplained infertility, Traditional East Asian medicine, Pregnancy, Network analysis, Data mining, Public Health, Standardization

## Abstract

**Background:**

This study aimed to investigate the prescription of traditional herbal medicines for the treatment of unexplained female infertility in Korea. By analyzing the relationships among the prescriptions and between the prescriptions and treatment outcomes, we aimed to confirm the utilization of standardized prescriptions and the effectiveness of this standardization.

**Methods:**

The data were derived from the “support projects” of the local government for infertile women to receive Korean medical treatments. The presciption data of 453 participants from 2017 to 2018 were analyzed. Data preprocessing, frequency analysis, and network analysis were conducted. For network analysis, the characteristics of the herbal medicine prescriptions were used to calculate the degree centrality, closeness centrality and eigenvector centrality. Modularity clustering was also performed for cluster analysis.

**Results:**

Fifty different prescriptions were used, among which only 22 were used for participants who became pregnant. The recommended standard prescriptions for support projects were used frequently. “*BaeranChacksangBang*” showed the highest level of in-degree centrality. Among the prescriptions for participants who became pregnant, “*JogyeongJongokTang*” and “*BaeranChacksangBang*” were the most influential nodes. “*BaeranChacksangBang*”*,* “*AnjeonYicheonTang*”*,* “*YukLinZu*” and “*JogyeongJongokTang*” had high closeness centrality among the prescriptions for participants who became pregnant. Clustering analysis of the prescriptions for participants who became pregnant revealed that the prescriptions showed the best modularity when divided into five groups.

**Conclusions:**

These findings depict the utilization of Korean herbal medicine in the real world and the dynamics underlying the herbal medicine prescription patterns for infertile women.

**Supplementary Information:**

The online version contains supplementary material available at 10.1186/s12906-023-04192-5.

## Background

Infertility is generally defined as the inability to conceive within one year without using contraception in individuals with normal sex life [1]. Approximately 15–30% of all patients with infertility have unexplained infertility with no specific abnormalities [2]. The American Society of Reproductive Medicine recommends that, as part of the standard infertility evaluation protocol, a semen test, an ovulation test, and hysterosalpingography are needed, and if necessary, an ovarian reserve test and laparoscopy should also be performed [2]. Unexplained female infertility is diagnosed if there are no abnormalities in the examination. There is no standardized treatment for unexplained infertility; therefore, it is treated empirically. The main treatment strategies include observational therapy based on the changes in the patients’ sexual life and lifestyle and assisted reproductive technologies, such as ovulation induction, artificial insemination, and in vitro fertilization [3].

Traditional East Asian Medicine (TEAM), particularly herbal medicine, has long been used to prevent and treat infertility, and several herbal medicines mentioned in classic books have been used as prescriptions for infertility. However, there are limitations in applying these prescriptions to infertile couples in modern society. The prescriptions from classic books, used thousands of years ago, might not be as effective in modern times, given the intervening fertility factors like the delayed marriage age and the significant changes in living environments from the past [4, 5]. Hence, a modern interpretation of herbal medicines and modifications of prescriptions are needed. Infertility treatment guidelines [6, 7] in Traditional East Asian Medicine (TEAM) that consider the current clinical environment have been published recently; however, studies on the real-world prescription of traditional medicines and their uses are still lacking. Therefore, it is necessary to study the current utilization of traditional medicine prescriptions to better reflect the current clinical status. In contrast, the therapeutic approaches to infertility in TEAM have the advantage of providing customized treatments based on the patient’s characteristics. Therefore, various prescription patterns are used in TEAM, even for a single diagnosis such as unexplained female infertility. The treatment regimens vary according to the constitution, age, and accompanying symptoms of the patient, which result in the addition or subtraction of herbs. However, in some cases, such variability may become an obstacle in standardizing treatments or ensuring medication safety.

In Korea, Traditional Korean Medicine (TKM) treatment for female infertility has been financially supported by the local government via “support projects”. These support projects provide medication guidelines to standardize prescriptions, while TKM doctors prescribe herbal medications at their discretion. While TKM doctors have the discretion to prescribe herbal medications, standardized prescriptions based on these guidelines can ensure both treatment effectiveness and safety. Therefore, it is necessary to objectively prove the validity of the recommended prescriptions to further promote standardization. Therefore, we aimed to evaluate the adherence to the prescription guidelines and utilization of herbal medications and identify the relationships between prescriptions and clinical results. Furthermore, since many TKM doctors use two different herbal medicine formulas for female infertility corresponding to before and after ovulation, we also aimed to identify the relationships between prescriptions to reveal combination patterns.

This study utilized anonymized data from the “Gyeonggi Korean Medicine Infertility Couple Support Project” [8, 9] to construct a herbal medicine prescription network and used network analysis to categorize the various prescriptions and examine the complex relationships between prescriptions and clinical results. This approach may help strengthen the standardization of prescriptions while confirming the outcome and effectiveness of prescription standardization. Understanding the actual utilization of herbal prescriptions will help TKM doctors identify optimal prescriptions in a real-world clinical setting.

## Methods

### Prescription data

The prescription data of the “Gyeonggi Korean Medicine Infertility Couple Support Project” [8, 9] from 2017 to 2018 were used. This project provided traditional medicine to women with unexplained infertility under the age of 44 years residing in Gyeonggi-do. The participants received acupuncture and moxibustion treatment for three months, in addition to receiving herbal medicines. The herbal medicine was administered six times for a duration of 15 days over a period of three months. The medication was discontinued if the patient conceived during the treatment period. If the subjects did not become pregnant during the 3-month treatment period, the pregnancy results were confirmed during an observation period of six months.

Although TKM doctors can freely prescribe herbal medicines, standardization has been attempted in the past through the recommendation of preferential uses for prescriptions in dedicated guidelines [6, 7]. It was recommended to consider each individual’s menstrual cycle phase when prescribing medication. It was recommended to start taking the follicular phase prescription on the third day of menstruation and administer the medication three times a day for 10 days, and thereafter, take the luteal phase prescription twice a day for 15 days. Table 1 lists the standard prescriptions suggested in the pre-training program for the TKM doctors participating in the study. Among the 536 women who participated in the project (276 in 2017 and 260 in 2018), 458 women completed their treatment course and had known pregnancy results. Among them, the prescription data for 453 women (228 in 2017 and 225 in 2018) were analyzed. The ingredients of the standard herbal medicine formulas are presented in Table 2.

**Table 1:** Herbal medicine guidelines for the Gyeonggi-do Korean Medicine infertility support project

2017 Herbal medicine guideline		
Pattern identification	Herbal medicine	
Kidney Deficiency	*YukLinZu, OnKyungTang*	
Qi Stagnation	*JogyeongJongokTang, KaewoolJongokTang*	
Dampness	*ChangbuDodamTang, OJeokSan*	
Blood Stasis	*GyejiBongnyeongHwan, HyulbuChukeoTang*	
Qi-Blood Deficiency	*GuiBiTang, PalJinTang*	
Damp-Heat	*HaedokSamulTang*	
2018 Herbal medicine guideline		
Pattern identification	Administration phase	Herbal medicine
Kidney Deficiency	Follicular phase	*YukLinZu*
Qi Stagnation		*JogyeongJongokTang*
Dampness		*ChangbuDodamTang*
Blood Stasis		*GyejiBongnyeongHwan*
Qi-Blood Deficiency		*OntoYuklinTang, GuiBiTang*
*-*	Luteal phase	*BaeranChacksangBang*^a^*, AnjeonYicheonTang*^b^*, TaesanBansucSan*^c^

^a^Prescription with ovulation and implantation enhancing effect

^b^Prescription to prevent miscarriage

^c^Prescription to treat habitual miscarriage

**Table 2:** Ingredients in the herbal medicine (HM) formulas

HM formula Korean name (Abbreviation)	Chinese Name^a^	Ingredients of the HM formula
*YukLinZu* (YLZ)	Yu Lin Zhu	Rehmanniae Radix Preparata, Cuscutae Semen**,** Cervi Cornu, Angelicae Gigantis Radix, Ginseng Radix, Atractylodis Rhizoma Alba, Poria Sclerotium, Paeoniae Radix, Eucommiae Cortex, Cnidii Rhizoma, and Glycyrrhizae Radix and Rhizoma
*OnKyungTang* (OKT)	Wen Jing Tang	Liriopis seu Ophiopogonis Tuber,Angelicae Gigantis Radix, Ginseng Radix, Pinelliae Tuber, Paeoniae Radix, Cnidii Rhizoma, Moutan Radicis Cortex, Asini Corii Colla, Glycyrrhizae Radix et Rhizoma, Evodiae Fructus, Cinnamomi Cortex, and Zingiberis Rhizoma Recens
*JogyeongJongokTang* (JJT)	Diao Jing Zhong Yu Tang	Rehmanniae Radix Preparata, Cyperi Rhizoma, Angelicae Gigantis Radix, Evodiae Fructus, Cnidii Officinale Rhizoma, Paeoniae Radix, Poria Sclerotium, Citri Unshius Pericarpium, Corydalis Tuber, Moutan Radicis Cortex, Zingiberis Rhizoma, Cinnamomi Cortex, Artemisiae Argyi Folium, and Zingiberis Rhizoma Recens
*KaewoolJongokTang* (KJT)	Kai Yu Zhong Yu Tang	Paeoniae Radix, Cyperi Rhizoma, Angelicae Gigantis Radix, Atractylodis Rhizoma Alba, Moutan Radicis Cortex,Poria Sclerotium, and Trichosanthes kirilowii
*ChangbuDodamTang* (CDT)	CangfuDaotan	Atractylodis Rhizoma, Cyperi Rhizoma, Aurantii Fructus Immaturus, Citri Unshius Pericarpium, Poria Sclerotium, Arisaematis Rhizoma, and Glycyrrhizae Radix and Rhizoma
*OJeokSan* (OJS)	Wu Ji San	Atractylodis Rhizoma, Ephedrae Herba, Citri Unshius Pericarpium, Magnoliae Cortex, Platycodonis Radix, Aurantii Fructus Immaturus, Angelicae Gigantis Radix, Zingiberis Rhizoma, Paeoniae Radix, Poria Sclerotium, Angelicae Dahuricae Radix, Cnidii Rhizoma, Pinelliae Tuber, Cinnamomi Cortex, Glycyrrhizae Radix et Rhizoma, Zingiberis Rhizoma Recens, and Allii Fistulosi Bulbus
*GyejiBongnyeongHwan* (GBH)	Gui Zhi Fu Ling Wan	Cinnamomi Ramulus, Poria Sclerotium, Moutan Radicis Cortex, Persicae Semen, and Paeoniae Radix
*HyulbuChukeoTang* (HCT)	Xue Fu Zhu Yu Tang	Persicae Semen, Angelicae Gigantis Radix, Rehmanniae Radix, Carthami Flos, Achyranthis Radix, Aurantii Fructus Immaturus, Paeoniae Radix Rubra, Platycodi Radix, Cnidii Rhizoma, Bupleuri Radix, and Glycyrrhizae Radix and Rhizoma
*GuiBiTang* (GBT)	Gui Pi Tang	Angelicae Gigantis Radix, Longanae Arillus, Zizyphi Spinosae Semen, Polygalae Radix, Ginseng Radix, Astragali Radix, Atractylodis Macrocephalac Rhizma, Poria Cocos, Aucklandiac Radix, Glycyrrhizae Radix, Zingiberis Rhizoma Recens, Jujubae Fructus, Gardeniae Fructus, and Bupleuri Radix
*PalJinTang* (PJT)	Ba Zhen Tang	Ginseng Radix, Atractylodis Rhizoma Alba, Poria Sclerotium, Glycyrrhizae Radix et Rhizoma, Rehmanniae Radix Preparata, Paeoniae Radix, Cnidii Rhizoma, and Angelicae Gigantis Radix
*HaedokSamulTang* (HST)	Jie Du Si Wu Tang	Phellodendri Cortex, Coptidis, Scutellariae Radix, Gardeniae Fructus, Angelicae Gigantis Radix, Cnidii Rhizoma, Paeoniae Radix, and Rehmanniae Radix
*OntoYuklinTang* (OYT)	Wen Tu Yu Lin Tang	Morindae Radix, Dioscoreae Rhizoma, Rubi Fructus, Atractylodis Rhizoma Alba, Ginseng Radix, and Massa Medicata Fermentata
*BaeranChacksangBang* (BCB)	Pailuan Zhao Chuang Fang	Cuscutae Semen, Dioscoreae Rhizoma, Rubi Fructus, Ginseng Radix, Lycii Fructus, Angelicae Gigantis Radix, Perillae Folium, Amomi Fructus, Artemisiae Argyi Folium, Zingiberis Rhizoma Recens, and Zizyphi Fructus
*AnjeonYicheonTang* (AYT)	An Dian Er Tian Tang	Ginseng Radix, Rehmanniae Radix Preparata, Atractylodis Rhizoma Alba, Dioscoreae Rhizoma, Corni Fructus, Glycyrrhizae Radix et Rhizoma, Eucommiae Cortex, Lycii Fructus, and Dolichoris Semen
*TaesanBansucSan* (TBS)	Taishan Panshi San	Ginseng Radix, Astragali Radix, Angelicae Gigantis Radix, Dipsaci Radix, Scutellariae Radix, Cnidii Rhizoma, Paeoniae Radix, Rehmanniae Radix Preparata, Atractylodis Rhizoma Alba, Glycyrrhizae Radix and Rhizoma, and Amomi Fructus

^a^Traditional Korean decoctions are named according to the Chinese pronunciation of the traditional Chinese characters

This study followed the guidelines of the Declaration of Helsinki and Tokyo for humans. This work was a retrospective study, and received a review exemption from the Institutional Review Board of the Dongguk University Ilsan Korean Medicine Hospital (2020–10-006). Written informed consent was obtained from all participants when they participated in the support project.

### Data preprocessing and analysis

A data preprocessing step was performed using Microsoft Excel 2019 (Microsoft corp., Redmond, WA, USA) to extract and classify meaningful words. In the case of herbal medicine prescriptions, prescription methods such as adding or subtracting some herbs from the original prescription or simultaneously combining different prescriptions were used. In some instances, multiple names referred to prescriptions with the same composition. Therefore, synonyms were unified into one word. When only the formulation method was different, multiple names were unified into one word and regarded as one prescription. When one or two herbs were added or subtracted from the original prescription, they were classified as the original prescription. In addition, prescription names that were considered to have been formulated by an individual TKM doctor were treated as “Others”. The classified prescription names were converted to English based on the Romanization of Korean and processed as abbreviations (Additional File 1).

The preprocessed data were analyzed using Microsoft Excel (Microsoft corp., Redmond, WA, USA) and IBM SPSS 23 (IBM corp., Armonk, NY, USA) for performing descriptive statistics analysis and frequency analysis for each prescription. In addition, the patterns of occurrence and associations among the medicines were analyzed using network analysis. Network analysis is used in various academic fields to understand complex relationships in which countless entities interact by simplifying them into networks of entities (nodes) and interactions (links). Centrality and cluster analyses were performed using Cyram NetMiner 4.0 (Cyram Inc., Seongnam, Korea) software. Through network analysis, the influence of each prescription and the connection state between the prescriptions and their characteristics were analyzed quantitatively and expressed visually. The herbal medicine combination network was constructed based on the proportion of co-occurrence among the herbal medicines. Centrality measures, such as node degree and betweenness, were calculated to identify the topologically important medicines, which are commonly referred to as hubs in the minimum spanning tree network. Modularity clustering was performed in cluster analysis [10, 11].

## Results

### Baseline characteristics of the patients

Tables 3 and 4 list the baseline characteristics of the 453 participants included in the study and their pregnancy outcomes, respectively. After the treatment, 49 participants conceived spontaneously. Among them, 11 participants experienced a miscarriage before 12 weeks. Among the 38 participants who maintained pregnancy up to 12 weeks, 28 deliveries were confirmed: 27 delivered singletons, and one delivered multiples in good health. The delivery results of 10 participants were not confirmed due to loss to follow-up.

**Table 3:** Characteristics of the participants (*N* = 453)

Variables	
Age (years)^a^	36.91 ± 4.07
Age of husband (years)^a^	38.61 ± 4.33
Height (cm)^a^	161.54 ± 4.97
Weight (kg)^a^	57.32 ± 9.18
Marriage duration (months)^a^	63.95 ± 38.48
Pregnancy trial duration (months)^a^	36.91 ± 4.08
Frequency of sexual intercourse (number/month)^a^	4.82 ± 2.76
Birth history	
Experienced full-term delivery^b^	44 (9.71%)
Experienced pre-term delivery^b^	8 (1.77%)
Experienced miscarriage^b^	165 (36.42%)
Previous treatment history	
Infertility treatment^b^	390 (86.09%)
Ovulation induction^b^	192 (42.38%)
IUI^b^	253 (55.85%)
IVF^b^	212 (46.80%)
Herbal medicine treatment^b^	214 (47.24%)
Male factor	
Sexually dysfunctioning partner^b^	22 (4.86%)
Abnormality in semen analysis^b^	98 (21.63%)

*IUI* Intrauterine insemination, *IVF *In vitro fertilization

^a^Mean ± standard deviation

^b^N (%)

**Table 4:** Pregnancy outcomes

Outcome	N (%)
Clinical pregnancy^a^	49 (10.82%)
Pregnancy maintenance (12 weeks)^b^	38 (8.39%)
Total	453 (100%)

^a^Presence of at least one gestational sac with the fetal heart rate confirmed by ultrasonography at 5–6 weeks of pregnancy

^b^Presence of fetal cardiac activity beyond 12 weeks of gestation

### Frequency analysis

An analysis of the frequency of each prescription showed that 50 prescriptions were used, excluding six “others”. Among them, 22 prescriptions and one “other” prescription were used for 49 participants who conceived. Tables 5 and 6 list the results of the frequency analysis for each prescription administered to participants and the pregnancy maintenance results.

**Table 5:** Frequency of prescriptions (*N* = 453)

Herbal medicine	Number of prescriptions
BCB	259
JJT	245
YLZ	113
GBT	68
TBS	57
AYT	52
CDT	45
OYT	24
OKT	17
GBH	11
SJT, PJT	7
SGT	6
OJS, HCT	4
SMT, BGT	3
GJE, DDT, HSS, HYT, ATE, YST, TJT, KJT	2
GST, WBT, JBT, WRT, ORS, CCT, DYJ, SS, GHT, SKT, PWS, GJT, PMT, JYT, YJT, JED, SYS, HYGT, PGT, DJT, OJCT, YM, SGJT, CYT, BHT	1
Others	6

*BCB BaeranChacksangBang*, *JJT JogyeongJongokTang*, *YLZ YukLinZu*, *GBT GuiBiTang*, *TBS TaesanBansucSan*, *AYT AnjeonYicheonTang*, *CDT ChangbuDodamTang*, *OYT OntoYuklinTang, OKT OnKyungTang*, *GBH GyejiBongnyeongHwan*, *SJT SipJeondaeboTang*, *PJT PalJinTang*, *SGT SeGungTang*, *OJS OJeokSan*, *HCT HyulbuChukeoTang*, *SMT SaMulTang*, *BGT BoGungTang*, *GJE GungguiJohyeolEum*, *DDT DoDamTang*, *HSS HyeongbangSabaekSan*, *HYT HyangsaYangyiTang*, *ATE AnTaeEum*, *YST YangkyukSanhwaTang, TJT TaeyeumJoweeTang*, *KJT KaewoolJongokTang*, *GST GyejibanhaSaenggangTang*, *WBT WonjamBoeumTang*, *JBT JeongsimBoeumTang, WRT WiRyeongTang, ORS OryeongSan, CCT CheongpoChugeoTang, DYJ DaeYoungJeon, SS SihoSogansan, GHT GungHaTang, SKT SaengkanKunbiTang*, *PWS PyungWiSan*, *GJT GamiJihwangTang*, *PMT PalMulTang*, *JYT JengjeongamiYijinTang*, *YJT YiJinTang*, *JED JeEumDan*, *SYS SoYoSan*, *HYGT HyangsaYukGunjaTang*, *PGT PalmulGunjaTang*, *DJT DokhwalJihwangTang*, *OJCT OgapiJangChukTang*, *YM YukMijihwangwon*, *SGJT SoGunJungTang*, *CYT CheongsimYeonjaTang*, *BHT BoHeoTang*

**Table 6:** Frequency of prescriptions for participants who became pregnant and pregnancy maintenance results

N^a^	Participants who became pregnant (*N* = 49)	Pregnancy maintenance results	
		Pregnancy maintenance for over 12 weeks (*N* = 38)	Early pregnancy loss before 12 weeks (*N* = 11)
BCB	27	23	4
JJT	27	19	8
YLZ	8	6	2
AYT	7	3	4
GBT	7	5	2
TBS	5	4	1
CDT	4	4	0
GBH	3	2	1
OKT	2	2	0
SJT	2	2	0
ATE	1	1	0
DDT	1	1	0
HCT	1	1	0
HYT	1	1	0
JED	1	1	0
JYT	1	1	0
PJT	1	1	0
SGT	1	1	0
SMT	1	1	0
SYS	1	1	0
YJT	1	1	0
YST	1	1	0
Others	1	1	0

*BCB BaeranChacksangBang*, *JJT JogyeongJongokTang*, *YLZ YukLinZu*, *AYT AnjeonYicheonTang*, *GBT GuiBiTang*, *TBS TaesanBansucSan*, *CDT ChangbuDodamTang*, *GBH GyejiBongnyeongHwan*, *OKT OnKyungTang*, *SJT SipJeondaeboTang*, *ATE AnTaeEum*, *DDT DoDamTang*, *HCT HyulbuChukeoTang*, *HYT HyangsaYangyiTang*, *JED JeEumDan*, *JYT JengjeongamiYijinTang*, *PJT PalJinTang*, *SGT SeGungTang*, *SMT SaMulTang*, *SYS SoYoSan*, *YJT YiJinTang*, *YST YangkyukSanhwaTang*

^a^Number of prescriptions

### Centrality analysis

#### Network visualization

Centrality analysis captures the role of the main prescription among all other prescriptions. The main prescription becomes a keyword with high connection centrality in the entire network and serves as a hub for a group of low keywords around this prescription. The thicker the link between the nodes, the higher the density and the stronger the relationship. A high density signifies that the node has strong connections with various other nodes. If the centrality of the connection is large, the size of the node is large. Moreover, the nodes that are used together exist in a relatively close position and are expressed as thick link lines [12].

**Fig. 1:**
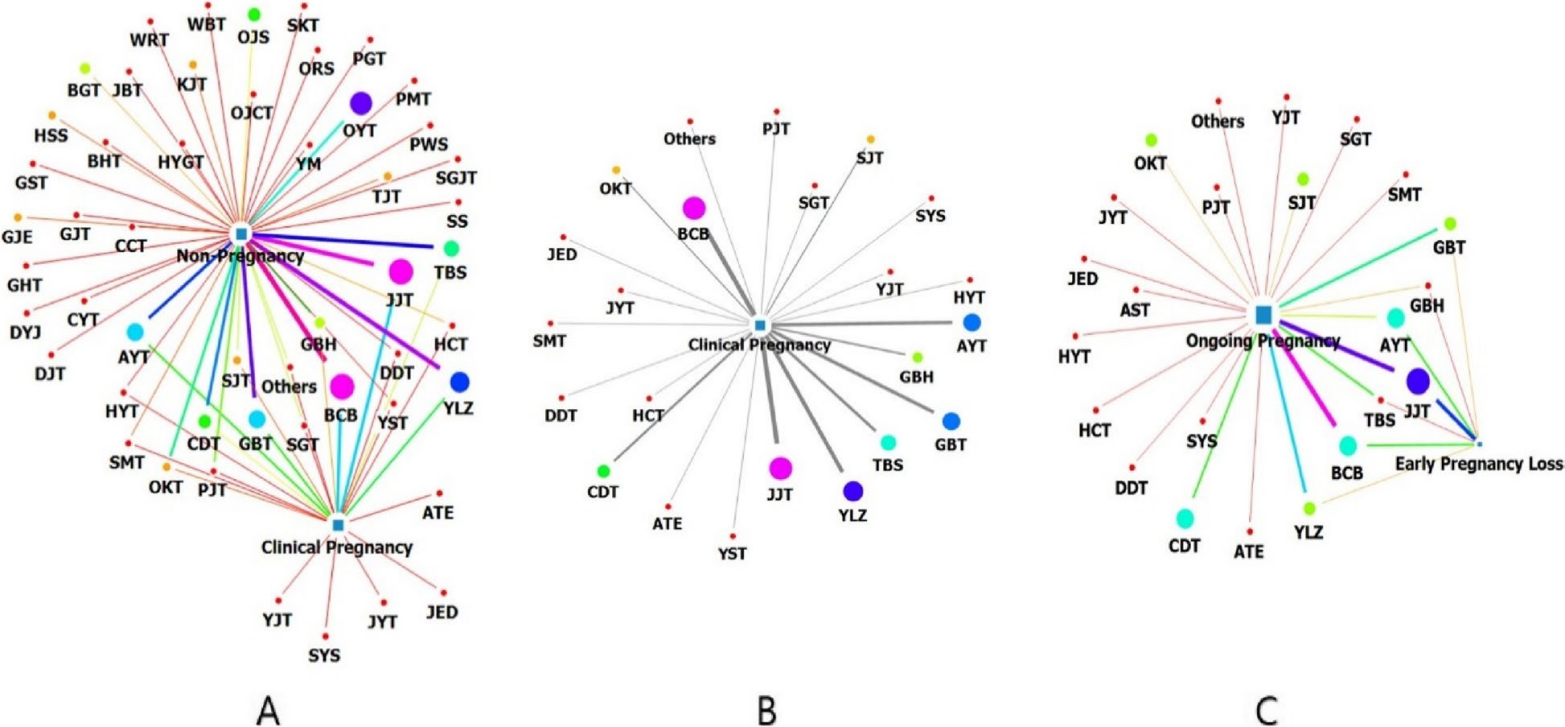
Network of the prescribed herbal medicines and pregnancy results. **a** JJT and BCB were the largest nodes, and they were the nodes most strongly linked with clinical pregnancy. **b** JJT and BCB were the largest nodes in the network of herbal medicines prescribed to participants who became pregnant. **c** BCB was the node most strongly linked with “ongoing pregnancy”. JJT: *JogyeongJongokTang*, AYT: *AnjeonYicheonTang*, BCB: *BaeranChacksangBang*, CDT: *ChangbuDodamTang*, GBT: *GuiBiTang*, YLZ: *YukLinZu*, TBS: *TaesanBansucSan*, GBH: *GyejiBongnyeongHwan*, Ongoing pregnancy: pregnancy maintained over 12 weeks, Early pregnancy loss: miscarriage before 12 weeks

Figure 1a presents a network map that visualizes the relationships between the pregnancy status and prescription used. In the case of the pregnant group, centering on “clinical pregnancy”, the density of the connecting links between *JogyeongJongokTang* (JJT, Diao Jing Zhong Yu Tang) and *BaeranChacksangBang* (BCB, Pailuan Zhao Chuang Fang) exhibited the thickest line, indicating that the nodes had the most active relationship. *YukLinZu* (YLZ, Yu Lin Zhu), *GuiBiTang* (GBT, Gui Pi Tang), *AnjeonYicheonTang* (AYT, An Dian Er Tian Tang), *TaesanBansucSan* (TBS, Taishan Panshi San), and *ChangbuDodamTang* (CDT, CangfuDaotan) showed strong connectivity, in decreasing order. *AnTaeEum* (ATE, An Tai Yin), *JeEumDan* (JED, JiYinDan), *JengjeongamiYijinTang* (JYT, Zhengchuan Jiawei Erchentang), *SoYoSan* (SYS, Xiao Yao San), and *YiJinTang* (YJT, Er Chen Tang) were not associated with the non-pregnant group; however, they were associated with the pregnant group.

In the non-pregnant group, thinner and smaller nodes were connected to “non-pregnancy” compared with the pregnant group. The links of BCB and JJT were also the thickest, followed by YLZ, GBT, TBS, AYT, and *OntoYuklinTang* (OYT, Wen Tu Yu Lin Tang) in decreasing order; CDT showed strong connectivity. Prescriptions including JJT, BCB, YLZ, GBT, AYT, TBS, and CDT showed a strong association in the non-pregnant and pregnant groups.

Figure 1b presents a network visualization map of the prescriptions administered to the pregnant group. The BCB and JJT nodes were larger than the other nodes and were used frequently. The next largest node was the YLZ node, followed by the GBT and AYT nodes. In addition, the BCB, JJT, JYT, YJT*, SeGungTang* (Xi Gong Tang), *HyulbuChukeoTang* (Xue Fu Zhu Yu Tang), and *GyejiBongnyeongHwan* (GBH, Gui Zhi Fu Ling Wan) nodes were located close to the “clinical pregnancy” node, suggesting that their connection with pregnancy was high.

Figure 1c presents the network relationship between whether the pregnancy was maintained for more than 12 weeks and the prescription. JJT had the largest node, followed by AYT, BCB, CDT, GBT, YLZ, *OnKyungTang* (OKT, Wen Jing Tang), and *SipJeondaeboTang* (Shi Quan Da Bu Tang*)*. In particular, the links appeared the thickest for BCB followed by JJT if the pregnancy was maintained for more than 12 weeks. Subsequently, it was confirmed that the prescriptions of YLZ, CDT, and GBT were connected with “ongoing pregnancy” by thick links in decreasing order. In the case of participants who became pregnant who could not continue with their pregnancy, JJT was connected with the thickest link, followed by AYT and BCB. JJT, AYT, and BCB were connected by thick links in participants who maintained and did not maintain pregnancy.

#### Degree centrality analysis

Most participants (90.07%) received two or more prescriptions rather than a single prescription during the treatment period. Forty-five patients (9.93%) received a single prescription. Therefore, this study investigated the relationships between the prescriptions used in the pregnant group by visualizing the degree centrality and the dosing frequency of each prescription. Degree centrality analysis is a method for finding nodes with high centrality [13]. The analysis identifies the number of nodes connected with a node and whether a specific node in the network is located at the center of the network. A node has a high degree of centrality if it has a large number of links connecting directly with other nodes. The closer the degree centrality is to 1, the more it is interpreted as being connected to many prescriptions; hence, its consideration as a core prescription. Table 7 lists the vector score of each prescription according to our degree centrality analysis. The vector ‘in/out’ means the direction of connection between nodes. The in-degree centrality indicates receiving more nominations from other nodes, and out-degree centrality indicates calling other nodes frequently. The in-degree centrality of BCB was highest at 0.227, indicating a high tendency to use other prescriptions when using BCB.

**Table 7:** Degree centrality of herbal medicines prescribed for participants who became pregnant

Herbal medicine	In-degree centrality	Out-degree centrality
BCB	0.227	0.136
AYT	0.182	0.136
JJT	0.136	0.227
YLZ	0.136	0.227
ATE	0.091	0.000
OKT	0.091	0.000
GBT	0.091	0.045
CDT	0.045	0.045
TBS	0.045	0.045
HCT	0.045	0.000
SJT	0.045	0.091
GBH	0.045	0.091
SMT	0.045	0.000
YJT	0.045	0.000
JYT	0.045	0.000
SGT	0.045	0.000
PJT	0.045	0.000
SYS	0.000	0.045
JED	0.000	0.182
DDT	0.000	0.136
HYT	0.000	0.045
YST	0.000	0.000
Others	0.045	0.000

*BCB BaeranChacksangBang*, *JJT JogyeongJongokTang*, *YLZ YukLinZu*, *AYT AnjeonYicheonTang*, *GBT GuiBiTang*, *TBS TaesanBansucSan*, *CDT ChangbuDodamTang*, *GBH GyejiBongnyeongHwan*, *OKT OnKyungTang*, *SJT SipJeondaeboTang*, *ATE AnTaeEum*, *DDT DoDamTang*, *HCT HyulbuChukeoTang*, *HYT HyangsaYangyiTang*, *JED JeEumDan*, *JYT JengjeongamiYijinTang*, *PJT PalJinTang*, *SGT SeGungTang*, *SMT SaMulTang*, *SYS SoYoSan*, *YJT YiJinTang*, *YST YangkyukSanhwaTang*

**Fig. 2:**
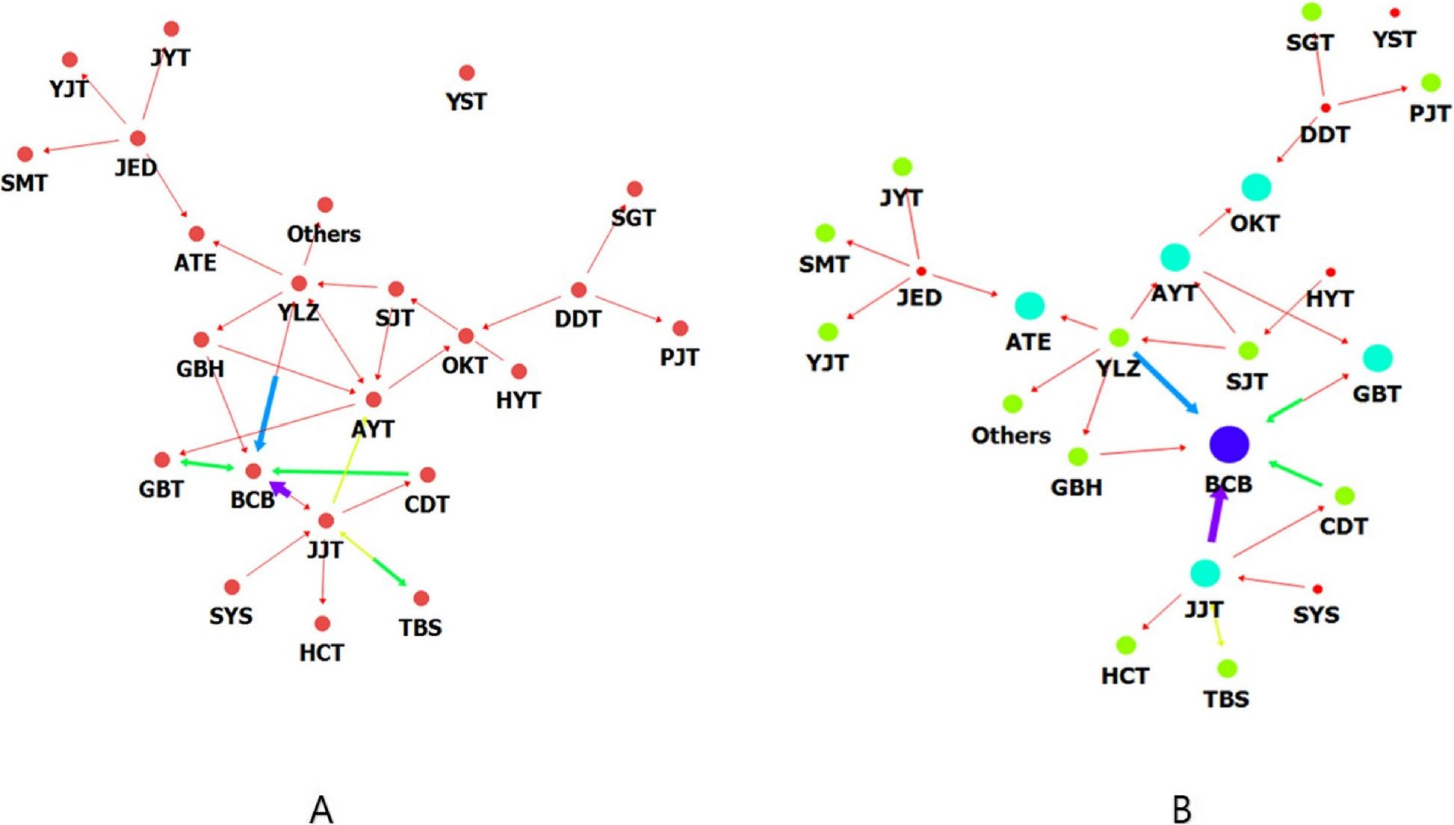
Degree centrality of herbal medicines prescribed for participants who became pregnant. **a** Degree centrality of herbal medicines prescribed for participants who became pregnant. The bold arrows were directed towards BCB, indicating high in-degree centrality. The arrow from JJT to BCB was especially thick. **b** Degree centrality of herbal medicine prescribed for participants who became pregnant who maintained pregnancy for over 12 weeks. The bold arrows were coming in towards BCB, and the arrow from JJT to BCB was especially thick. BCB: *BaeranChacksangBang*, JJT: *JogyeongJongokTang*

The network visualization map of the degree centrality analysis (Fig. 2a) showed many arrows pointing to the nodes of BCB, AYT, JJT, and YLZ, which had relatively high in-degree centrality. In particular, two prescriptions, namely BCB and JJT, tended to be used together. Degree centrality analysis of participants who were pregnant for more than 12 weeks (Fig. 2b) showed that JJT was heading towards BCB with a thick link.

#### Closeness centrality analysis

**Fig. 3:**
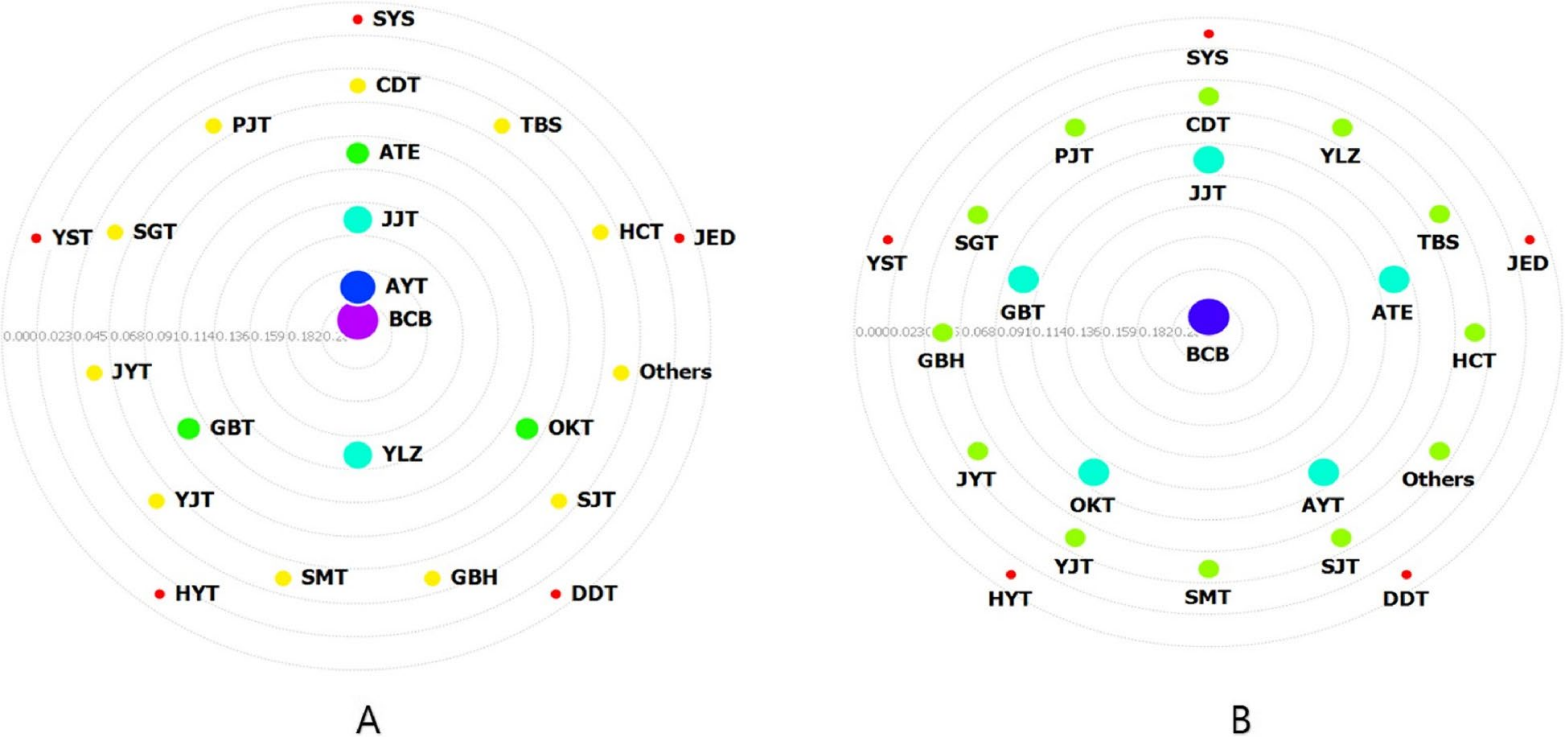
Closeness centrality of the herbal medicines prescribed for participants who became pregnant. **a** Closeness centrality of the herbal medicines prescribed for participants who became pregnant. BCB was located at the center of the network. **b** Closeness centrality of the herbal medicines prescribed for participants who became pregnant who maintained pregnancy for over 12 weeks. BCB was located at the center. BCB: *BaeranChacksangBang*, JJT: *JogyeongJongokTang*, GBT: *GuiBiTang*, ATE: *AnTaeEum*, OKT: *OnKyungTang*, AYT:* AnjeonYicheonTang*

Closeness centrality measures the centrality within a network by considering the indirect and direct connections between the nodes [14, 15]. Figure 3a presents the results of the closeness centrality analysis of the prescriptions administered to participants who became pregnant. Among the prescriptions for participants who became pregnant, BCB, AYT, YLZ, and JJT were centrally located in the entire network. According to the results of the closeness centrality analysis of pregnant patients who maintained pregnancy for more than 12 weeks, BCB was located at the center and surrounded by JJT, GBT, ATE, AYT, and OKT (Fig. 3b).

#### Eigenvector centrality analysis

Eigenvector centrality is useful for finding the most influential central node in a network [16]. This reflects the view that the nodes connected to nodes with a high influence have higher centrality than the nodes connected to nodes with a low influence [12]. In a network, if a node influences other nodes, those nodes continue to influence other nodes; the first node can be said to have the greatest influence [12]. Therefore, the eigenvector centrality of a node is calculated as the sum of the constant part, which is the original centrality value of the node, the derived part, which is derived from the centrality value of other nodes connected to this node, and the reflected part, which is the centrality value returned from another node [17]. Table 8 lists the eigenvector value of each prescription. BCB and JJT had exceptionally high values. Thus, BCB and JJT were highly influential and received considerable influence from those around them.

**Table 8:** Eigenvector centrality of the herbal medicines prescribed for participants who became pregnant

Herbal medicine	Eigenvector centrality^a^	Reflected part	Derived part	Constant part
BCB	0.677	0.248	0.396	0.033
JJT	0.643	0.226	0.385	0.033
YLZ	0.195	0.018	0.145	0.033
CDT	0.178	0.016	0.129	0.033
GBT	0.143	0.010	0.100	0.033
TBS	0.128	0.009	0.087	0.033
AYT	0.114	0.006	0.076	0.033
GBH	0.066	0.003	0.030	0.033
HCT	0.043	0.002	0.008	0.033
SYS	0.043	0.002	0.008	0.033
SJT	0.021	0.002	-0.014	0.033
ATE	0.013	0.002	-0.022	0.033
OKT	0.008	0.002	-0.027	0.033
HYT	0.001	0.002	-0.034	0.033
JED	0.001	0.003	-0.035	0.033
DDT	0.001	0.003	-0.035	0.033
SMT	0.000	0.002	-0.035	0.033
YJT	0.000	0.002	-0.035	0.033
JYT	0.000	0.002	-0.035	0.033
SGT	0.000	0.002	-0.035	0.033
PJT	0.000	0.002	-0.035	0.033
YST	0.000	0.002	-0.035	0.033
Others	0.013	0.002	-0.022	0.033

*BCB BaeranChacksangBang*, *JJT JogyeongJongokTang*, *YLZ YukLinZu*, *AYT AnjeonYicheonTang*, *GBT GuiBiTang*, *TBS TaesanBansucSan*, *CDT ChangbuDodamTang*, *GBH GyejiBongnyeongHwan*, *OKT OnKyungTang*, *SJT SipJeondaeboTang*, *ATE AnTaeEum*, *DDT DoDamTang*, *HCT HyulbuChukeoTang*, *HYT HyangsaYangyiTang*, *JED JeEumDan*, *JYT JengjeongamiYijinTang*, *PJT PalJinTang*, *SGT SeGungTang*, *SMT SaMulTang*, *SYS SoYoSan*, *YJT YiJinTang*, *YST YangkyukSanhwaTang*

^a^Eigenvector centrality = Reflected part + Derived part + Constant part

### Cluster analysis

**Fig. 4:**
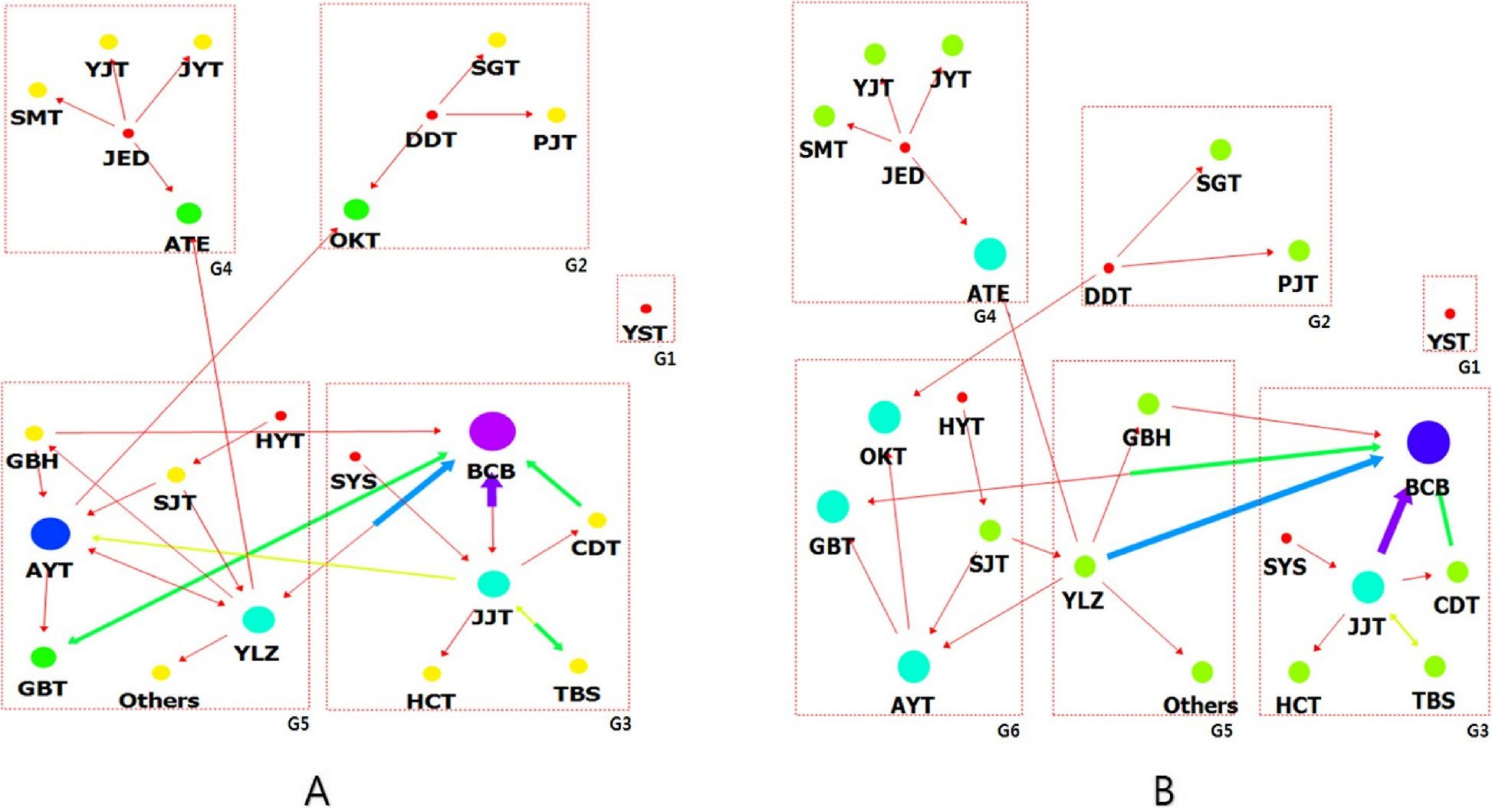
Clustering analysis results of the herbal medicines prescribed for participants who became pregnant. **a** Clustering results of the herbal medicines prescribed for participants who became pregnant. The prescriptions were classified into five groups. **b** Clustering results of the herbal medicines prescribed for participants who became pregnant who maintained pregnancy for over 12 weeks. The prescriptions were classified into six groups. YST: *YangkyukSanhwaTang*, BCB: *BaeranChacksangBang*, JJT: *JogyeongJongokTang*, ATE: *AnTaeEum*, YLZ: *YukLinZu*, AYT: *AnjeonYicheonTang*, OKT: *OnKyungTang*, GBT: *GuiBiTang*

Modularity clustering was performed to visualize the network patterns and connection relationships that all prescriptions constituted with each other, focusing on the prescriptions used for participants who became pregnant [18, 19]. Consequently, five groups were formed: G1, G2, G3, G4, and G5 (Fig. 4a). The node size is expressed through the centrality index, indicating the relative importance between the nodes. A larger size indicates higher importance. An analysis of the group composition centering on large nodes revealed the following: G2 consisted of OKT; G3 consisted of BCB and JJT; G4 consisted of ATE; and G5 consisted of AYT, YLZ, and GBT. The prescriptions used in the participants whose pregnancy was maintained for more than 12 weeks led to a similar group formation (Fig. 4b). The highest modularity was observed when there were six groups. At this time, although G1, G3, and G4 were the same as those in Fig. 4a, the other groups were subdivided into small groups.

## Discussion

### Summary of the main results

This study analyzed the data derived from the Gyeonggi-do Korean Medicine support project for infertile couples from 2017 to 2018. The prescription data of 453 female participants were analyzed. Fifty different prescriptions were used, except “others”, but only 22 were used in participants that conceived. Among the 50 prescriptions, those that were recommended during the pre-training period represented relatively large nodes, confirming that prescription standardization efforts are being reflected in the clinical field.

The relationships between “prescriptions”, “pregnancy”, and “pregnancy maintenance” were visualized through two-mode analysis to verify the effectiveness of the recommended prescriptions. All recommended prescriptions, except for OYT, had a close relationship with the pregnant group. The standard recommended prescriptions, including JJT, BCB, YLZ, GBT, AYT, TBS, and CDT, were used frequently regardless of a positive pregnancy result or pregnancy maintenance.

When confirming the frequency of the prescriptions depending on whether or not they helped maintain pregnancy for 12 weeks in the participants who became pregnant, JJT, GBT, BCB, AYY, YLZ, TBS, and GBH were used frequently in both groups. These prescriptions were standard recommended prescriptions that were used frequently, regardless of whether the participants were pregnant. AYT, BCB, and TBS are prescriptions for preventing miscarriage. Accordingly, it is assumed that they were selected for and administered to the participants judged to be at a high risk of pregnancy loss. In the prescription network map of participants who maintained pregnancy for more than 12 weeks, the links between pregnancy maintenance and the BCB and JJT nodes were thick. This demonstrated that BCB and JJT had a close connection with pregnancy maintenance.

According to the degree centrality analysis of the prescriptions for participants who became pregnant, BCB, AYT, JJT, and YLZ had high in-degree centrality. Therefore, these prescriptions were often used in combination with other prescriptions. In particular, BCB had the highest in-degree centrality. BCB is prescribed to sustain pregnancy and prevent miscarriage. For those post-ovulation, it’s used as a foundational prescription, considering the potential for pregnancy to ensure the selection of safer medications. Closeness centrality analysis showed that among the prescriptions used for participants who became pregnant, BCB, AYT, YLZ, and JJT had a strong influence on the network.

In addition, eigenvector centrality analysis showed that among the prescriptions used during pregnancy, BCB and JJT were highly influential and also influenced by their own surroundings. The prescriptions of BCB and JJT influenced each other (Table 8). Therefore, these two prescriptions could be prescribed in a single combination.

Lastly, cluster analysis using the nodes prescribed to the participants who became pregnant showed the best modularity when classified into five groups (Fig. 4). G1 consisted of YST, and when looking at the large nodes in the remaining groups, G2 consisted of OKT; G3 consisted of BCB and JJT; G4 contained ATE; and G5 consisted of AYT, YLZ, and GBT.

### Agreement with other studies

In infertility, herbal medicine is used as a primary treatment along with acupuncture and moxibustion. Existing clinical studies on herbal medicine treatment for infertility with unknown causes have usually been conducted using one or two specific prescriptions. It is difficult to analyze the results from the use of various prescriptions in actual clinical sites. There have been few studies on the current status of Korean herbal medicine utilization for managing female infertility in clinical practice. There was one previous study based on a questionnaire survey [20], studies that analyzed the data from only one [21] or several TKM clinics [22], and a study that analyzed the herbal medicines most frequently selected as basic prescriptions by the support projects of the local government [23]. In the latter, the data of the support projects from more than 100 TKM clinics participating each year were used. The support projects determined the criteria for selecting participants, but the treatment was performed similarly as in actual clinical treatment. Therefore, the prescription contents and results in the actual clinical sites could be confirmed.

Several previous studies were related to the infertility Korean medicine treatment support projects; however, there have been very few studies on the actual application status of the prescriptions recommended in the projects. One previous study analyzed the characteristics and outcomes of local support projects and the status of herbal medicine utilization [23].

Therefore, in this study, network analysis was used to evaluate the relationships between the prescriptions and between the prescriptions and treatment outcomes, to confirm the utilization of standardized prescriptions, and to explore the effectiveness of this standardization. This study is clinically significant because it analyzed real-world data, determined the application status of prescription recommendations, and evaluated the results of unexplained female infertility treatment.

### Potential mechanisms and implications for research

JJT and BCB were the most frequently used combinations, and they were closely associated with pregnancy. These results are consistent with those of previous studies that found that JJT was the most frequently used prescription [20]. JJT is a popular prescription mainly used for menstrual disorders and female infertility, and has been studied in several case reports [24–26] and experimental studies [27, 28]. BCB is a prescription for ovulation and implantation. This prescription was designed by Kim using *YukLinZu*, *OntoYukLinZu*, and *SuTaeHwan* [29]. BCB was used as a medication in clinical trials for unexplained female infertility, and its effectiveness and safety were evaluated [30]. JJT and BCB were the main prescriptions in the guidelines for the support projects. As shown in this study, the standardization efforts based on the use of guidelines were well-reflected in the clinical field, and the results were positive. Nevertheless, further studies on the two main prescriptions, JJT and BCB, are needed to confirm their effectiveness and safety.

Recently, as the need for standardization has emerged in the traditional medical field, clinical treatment guidelines and clinical pathways have been developed and applied. This has helped improve the quality of medical care and provide predictable medical services to increase patient and medical staff satisfaction and provide cost-effective treatment. This work can be regarded as evidence-based standardization efforts and can serve as an opportunity to convert Korean medicine, which has the distinct characteristic of providing customized treatments, into standardized medicine. Specifically, in the infertility treatment realm, a guide was produced for the standardization of Korean medicine treatments and applied to the infertility support projects.

In general, the guidelines for Korean medicine clinical practice are developed through expert agreement based on the results of published clinical research studies. The number of clinical studies on Korean medicine for infertility is very small. In addition, there are many differences between actual clinical practice where various prescriptions are typically used and clinical trials most often using one or two medicines. Therefore, this study was conducted to assess the extent to which the actual clinical field data reflected the guidelines. In turn, practical guidelines reflecting the real world can be developed if network analysis is performed based on the medical records collected at the clinical sites and used as a reference. Based on the study results, when using a single prescription for infertility treatment considering the recommended standard prescription for infertility treatment and the actual prescription utilization and clinical results, JJT and BCB combinations can be recommended first. In addition, when applying prescriptions according to the patterns of infertility, the following combinations can be recommended: JJT and BCB, YLZ and BCB or AYT, GBT and BCB, CDT and BCB, and GBH and BCB.

### Limitations

Care should be taken when generalizing the results of this study, as the source material used in the analysis was limited to the data of a specific project program. In order to overcome this limitation, it will be necessary to accumulate prescription data from infertility treatments for a long period of time and proceed with large-scale data analysis research. Medications for infertility require continuous monitoring for their safety over a long period of time because they are prescribed to those who plan to become pregnant or who could potentially conceive. To ensure reproductive safety, data from long-term follow-up is necessary. Moreover, the types of prescriptions used for one participant varied from one to a maximum of five, and each one of these was analyzed in the same way. Further research is warranted to improve the prescription guidelines for infertility support projects and to strengthen the supporting evidence.

## Conclusions

We analyzed the clinical data from traditional Korean herbal medicine treatments for unexplained female infertility. Although various prescriptions were used in the clinical field, the prescriptions for participants who became pregnant were limited. In addition, standard prescriptions were mainly used, and they were closely related to pregnancy. In particular, BCB and JJT had a strong relationship with 12-week pregnancy maintenance, and the two prescriptions strongly influenced each other. We were able to confirm that standardization according to the guidelines was well applied in the clinical field. The results of this study provide preliminary clinical evidence supporting the development of standard prescription guidelines in consideration of its current utilization in actual clinical practice and pregnancy results. Thus, further research is needed to explore the effectiveness and underlying mechanisms of traditional Korean herbal medicine for unexplained female infertility.

### Supplementary Information

**Additional file 1.** List of Abbreviations.

## Data Availability

The data that support the findings of this study are available from the corresponding author upon request.

## Abbreviations

TEAM: Traditional East Asian Medicine
TKM: Traditional Korean Medicine
JJT: *JogyeongJongokTang*
BCB: *BaeranChacksangBang*
YLZ: *YukLinZu*
GBT: *GuiBiTang*
AYT: *AnjeonYicheonTang*
TBS: *TaesanBansucSan*
CDT: *ChangbuDodamTang*
ATE: *AnTaeEum*
JYT: *JengjeongamiYijinTang*
YJT: *YiJinTang*
OYT: *OntoYuklinTang*
GBH: *GyejiBongnyeongHwan*
OKT: *OnKyungTang*
Clinical pregnancy: Presence of at least one gestational sac with the fetal heart rate confirmed by ultrasonography, at 5–6 weeks of pregnancy
Ongoing pregnancy: Presence of fetal cardiac activity beyond 12 weeks of gestation
