# Supplementary material for: Utilization of traditional herbal medicine formulas for unexplained female infertility in Korea: a retrospective study
Source: BMC Complement Med Ther. 2023 Oct 19;23:369. doi: 10.1186/s12906-023-04192-5 (PMC10585731; doi:10.1186/s12906-023-04192-5)
Supplement: Supplementary file 1 — Additional file 1. List of Abbreviations. [file 12906_2023_4192_MOESM1_ESM.docx]

**Supplementary Table 1. List of Abbreviations**

| Herbal Medicine (Korean name) | Abbreviation | Chinese name^a^ |
| --- | --- | --- |
| *AnTaeEum* | ATE | An Tai Yin |
| *AnjeonYicheonTang* | AYT | An Dian Er Tian Tang |
| *BaeranChacksangBang* | BCB | Pailuan Zhao chuang Fang |
| *BoGungTang* | BGT | BoGungTang |
| *BoHeoTang* | BHT | BuXueTang |
| *CheongpoChugeoTang* | CCT | Qingbaozhuyutang |
| *ChangbuDodamTang* | CDT | CangfuDaotan |
| *CheongsimYeonjaTang* | CYT | QingXin Lian Zi tang |
| *DoDamTang* | DDT | Dao Tan Tang |
| *DokhwalJihwangTang* | DJT | DokhwalJihwangTang |
| *DaeYoungJeon* | DYJ | Da Ying Jian |
| *GyejiBongnyeongHwan* | GBH | GuiZhi Fu Ling Wan |
| *GuiBiTang* | GBT | Gui Pi Tang |
| *GyejibanhaSaenggangTang* | GST | GuiZhi BanXia ShengJiang Tang |
| *GungHaTang* | GHT | GungHaTang |
| *GungguiJohyeolEum* | GJE | GungguiJohyeolEum |
| *GamiJihwangTang* | GJT | Modified Dihuang Decoction |
| *HyulbuChukeoTang* | *HCT* | Xue Fu Zhu Yu Tang |
| *HyeongbangSabaekSan* | *HSS* | HyeongbangSabaekSan |
| *HyangsaYukGunjaTang* | *HYGT* | Xiang Sha Liu Jun Zi Tang |
| *HyangsaYangyiTang* | *HYT* | Xiang Sha Yang Wei Tang |
| *JeongsimBoeumTang* | *JBT* | JeongsimBoeumTang |
| *JeEumDan* | *JED* | JiYinDan |
| *JogyeongJongokTang* | *JJT* | Diao Jing Zhong Yu Tang |
| *JengjeongamiYijinTang* | *JYT* | Zhengchuan Jiawei Erchentang |
| *KaewoolJongokTang* | *KJT* | Kai Yu Zhong Yu Tang |
| *OgapiJangChukTang* | *OJCT* | Wujiapi ZhuangJi Tang |
| *OJeokSan* | *OJS* | Wu Ji San |
| *OnKyungTang* | *OKT* | Wen Jing Tang |
| *ORyeongSan* | *ORS* | Wu Ling San |
| *OntoYuklinTang* | *OYT* | Wen Tu Yu Lin Tang |
| *PalmulGunjaTang* | *PGT* | PalmulGunjaTang |
| *PalMulTang* | *PMT* | BawuTang |
| *PalJinTang* | *PJT* | Ba Zhen Tang |
| *PyungWiSan* | *PWS* | Ping Wei San |
| *SoGunJungTang* | *SGJT* | Si Jun Zi Tang |
| *SeGungTang* | *SGT* | SeGungTang |
| *SipJeondaeboTang* | *SJT* | Shi Quan Da Bu Tang |
| *SaengkanKunbiTang* | *SKT* | SaengkanKunbiTang |
| *SaMulTang* | *SMT* | Si Wu Tang |
| *SihoSogansan* | *SS* | Chai Hu Shu Gan San |
| *SoYoSan* | *SYS* | Xiao Yao San |
| *TaesanBansucSan* | *TBS* | TaesanBansucSan |
| *TaeyeumJoweeTang* | *TJT* | TaeyeumJoweeTang |
| *WonjamBoeumTang* | *WBT* | WonjamBoeumTang |
| *WiRyeongTang* | *WRT* | Wei Ling Tang |
| *YiJinTang* | *YJT* | Er Chen Tang |
| *YukLinZu* | *YLZ* | Yu Lin Zhu |
| *YukMijihwangwon* | *YM* | Liuwei dihuang wang |
| *YangkyukSanhwaTang* | *YST* | Liang Ge San Huo Tang |

^a^Traditional Korean decoctions are named according to the Chinese pronunciation of the traditional Chinese characters
